# Karyotype complexity and prognosis in acute myeloid leukemia

**DOI:** 10.1038/bcj.2015.114

**Published:** 2016-01-15

**Authors:** F Stölzel, B Mohr, M Kramer, U Oelschlägel, T Bochtler, W E Berdel, M Kaufmann, C D Baldus, K Schäfer-Eckart, R Stuhlmann, H Einsele, S W Krause, H Serve, M Hänel, R Herbst, A Neubauer, K Sohlbach, J Mayer, J M Middeke, U Platzbecker, M Schaich, A Krämer, C Röllig, J Schetelig, M Bornhäuser, G Ehninger

**Affiliations:** 1 Medizinische Klinik und Poliklinik I, Universitätsklinikum Carl Gustav Carus der TU Dresden, Dresden, Germany; 2 Abteilung Innere Medizin V, Universität Heidelberg, Heidelberg, Germany; 3 Medizinische Klinik A, Universitätsklinikum Münster, Münster, Germany; 4 Robert Bosch Hospital, Stuttgart, Germany; 5 Charité–Universitätsmedizin Berlin, Berlin, Germany; 6 Asklepios Klinik St Georg, Hamburg, Germany; 7 Asklepios Klinik St Georg, Hamburg, Germany; 8 Medizinische Klinik und Poliklinik II, Universitätsklinikum Würzburg, Würzburg, Germany; 9 Medizinische Klinik 5, Universitätsklinikum Erlangen, Erlangen, Germany; 10 Medizinische Klinik II, Klinikum der J.W. Goethe Universität, Frankfurt, Germany; 11 Klinik für Innere Medizin III, Klinikum Chemnitz, Chemnitz, Germany; 12 Kliniken für Innere Medizin, Hämatologie/Onkologie und Immunologie, Universitätsklinikum Marburg, Marburg, Germany; 13 University Hospital Brno, Brno, Czech Republic; 14 Klinik für Hämatologie und Onkologie, Rems-Murr-Kliniken, Winnenden, Germany

## Abstract

A complex aberrant karyotype consisting of multiple unrelated cytogenetic abnormalities is associated with poor prognosis in patients with acute myeloid leukemia (AML). The European Leukemia Net classification and the UK Medical Research Council recommendation provide prognostic categories that differ in the definition of unbalanced aberrations as well as the number of single aberrations. The aim of this study on 3526 AML patients was to redefine and validate a cutoff for karyotype complexity in AML with regard to adverse prognosis. Our study demonstrated that (1) patients with a pure hyperdiploid karyotype have an adverse risk irrespective of the number of chromosomal gains, (2) patients with translocation t(9;11)(p21∼22;q23) have an intermediate risk independent of the number of additional aberrations, (3) patients with ⩾4 abnormalities have an adverse risk *per se* and (4) patients with three aberrations in the absence of abnormalities of strong influence (hyperdiploid karyotype, t(9;11)(p21∼22;q23), CBF-AML, unique adverse-risk aberrations) have borderline intermediate/adverse risk with a reduced overall survival compared with patients with a normal karyotype.

## Introduction

The term *complex aberrant* is designated to describe karyotypes with multiple unrelated cytogenetic abnormalities. In acute myeloid leukemia (AML), 10–14% of all AML patients, and up to 23% among older AML patients, have karyotypes with ⩾3 aberrations.^1, 2, 3, 4^ These karyotypes with ⩾3 aberrations are classified as adverse genetic risk according to the recommendations of the European Leukemia Net (ELN).^1^ However, the UK National Cancer Research Institute Adult Leukaemia Working Group (abbreviated as MRC for Medical Research Council) classification requires ⩾4 abnormalities as an informative cutoff for adverse prognosis.^5^ Beyond the 3 vs 4 cutoff discordance, the impact of the so-called pure hyperdiploid karyotype (HDK) without structural aberrations or monosomies has not been addressed conclusively.^6, 7^ Further complicating, the definition of unique adverse-risk abnormalities, which define adverse risk *per se*, is not fully congruent in both classification systems, with some abnormalities conferring adverse risk according to the ELN but not the MRC and vice versa. Given this heterogeneity, further exploration of complexity seems desirable for several reasons. First, it has been demonstrated that in the adverse-risk group, some patients with certain chromosomal abnormalities fare even worse than others when receiving standard treatment regimens for adverse-risk patients.^8, 9, 10, 11, 12^ Second, better individual risk prognostication and uniformly defined adverse-risk group allocation are required in order to homogeneously compare treatment regimens at different institutions.

The aim of this study was to define the optimized cutoff of complexity in adult AML in the context of the number of unrelated aberrations (3 vs ⩾4) as well as to define the impact of the pure HDK within these groups. Therefore, we evaluated the survival of 417 intensively treated adult non-APL and non CBF-AML patients with complex aberrant karyotypes out of 3526 AML patients who were included in three prospective, randomized, multicenter treatment trials of the Study Alliance Leukemia.

## Patients and methods

### Patient population

The databases of three prospective, randomized trials of the Study Alliance Leukemia, which enrolled a total of 3526 non-APL, intensively treated AML patients between February 1996 and November 2009, were reviewed for patients with multiple cytogenetic aberrations (⩾3) as well as normal karyotype (NK as a control group). The studies were approved by the institutional review boards of all participating centers of the Study Alliance Leukemia in agreement with the Declaration of Helsinki and registered with the National Clinical Trial numbers 00180115 (AML96 trial), 00180102 (AML2003 trial) and 00180167 (AML60+ trial). Written informed consent had been obtained from each patient.

At diagnosis, chromosome analyses were performed on bone marrow and/or peripheral blood samples using standard techniques, including short-term cultures as reported recently.^13^ Karyotype description was performed in accordance with the International System for Human Cytogenetic Nomenclature criteria.^14^ According to the definition of the MRC, a balanced translocation, for example, t(8;21)(q22;q22), was defined as a single abnormality, because the two breaks and fusions lead to one active chimeric fusion protein. A balanced translocation involving more than two chromosomes was also regarded as a single abnormality. Trisomies or monosomies were regarded as single abnormalities, whereas the gain of two chromosomes, even if they were identical (e.g., tetrasomy 8), was regarded as two abnormalities. Unbalanced translocations leading to gain and loss of chromosomal material were counted as two abnormalities.^5^ For instance, a derivative chromosome der(7)t(1;7)(q21;q22) is characterized by a partial monosomy 7q as well as a partial trisomy 1q. In this manner, an isochromosome i(17)(q10) results in two aberrations, that is, monosomy 17p and trisomy 17q. The monosomal karyotype (MK) was defined by the presence of two or more distinct autosomal chromosome monosomies or a single autosomal chromosome monosomy in the presence of one or more structural chromosomal abnormalities.^8^

### Cytogenetic definitions

Out of the 3526 patients, a total of 2007 patients with either a complex karyotype or a normal karyotype were identified for further analyses (*n*=1590 patients with NK; *n*=417 patients with ⩾3 aberrations which accounted for 30% of the patients in the AML96 trial and 29% of the patients in the AML2003/60+ trials—referring to those patients for whom an aberrant karyotype was diagnosed). Patient characteristics are summarized in Tables 1A and B. The median follow-up time for all patients was 6.2 years (interquartile range, 4.5–8 years). Core-binding factor AML patients (CBF-AML, t(8;21)(q22;q22), inv(16)(p13q22), t(16;16)(p13:q22)) were excluded since additional chromosomal abnormalities even if they resulted in complex aberrant karyotypes have no or little impact on the outcome of patients with favorable-risk CBF-AML^5^ and could be confirmed with our CBF-AML patients.^11, 15^ However, previous results demonstrated an independent influence of the pure HDK on patients' outcome worsening overall survival (OS) and event-free survival significantly.^15^ The scoring criterion for pure HDK performed in our analyses was defined by (i) gains of whole chromosomes (e.g., trisomies, tetrasomies), (ii) no additional structural aberrations and (iii) no monosomies.

The following distinct cytogenetic features were included as possible candidates of strong influence: (I) three or four unrelated aberrations, (II) specific adverse-risk aberrations that induce an adverse outcome *per se*: unique adverse-risk aberrations defined by the ELN and the MRC were applied in this study which were, in detail, inv(3)(q21q26), t(3;3)(q21;q26), abnl(3q) except t(3;5)(q21∼25;q31∼q35), −5, del(5q), add(5q), −7, del(7q), add(7q), t(6;9)(p23;q34), t(v;11)(v;q23) except t(9;11)(p21∼22;q23), −17 and abnl(17p),^1, 5^ (III) AML with recurrent genetic abnormalities according to the World Health Organization. This category includes the recurrent abnormalities t(9;11)(p22;q23), t(6;9)(p23;q34), inv(3)(q21q26.2)/t(3;3)(q21;q26.2), and t(1;22)(p13;q13).^16^ Translocation t(6;9) and inv(3)/t(3;3) are specific adverse-risk aberrations and therefore already included in that category. Translocation t(1;22) is a rare aberration with *n*=1 patient. Therefore, no further investigation was possible. Thus, only t(9;11) remained as a feature of particular interest, and (IV) pure HDK with gains of whole chromosomes (e.g., trisomies, tetrasomies), but without additional structural aberrations or monosomies.

**Figure 1:**
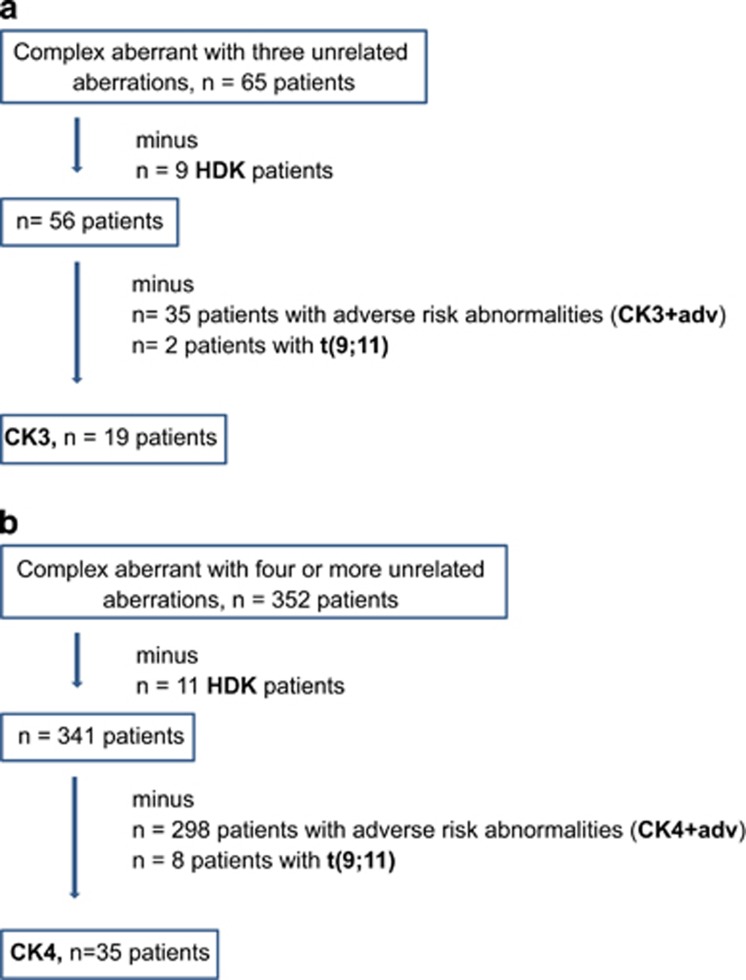
The flowcharts depicts all patients characterized by 3 (**a**) and ⩾4 unrelated abnormalities (**b**), respectively, and end with the groups CK3 and CK4. Patients with (i) pure hyperdiploid karyotype (HDK), (ii) adverse-risk aberrations and (iii) t(9;11) were removed constituting distinct groups because of their strong specific influence on outcome *per se*. All of the complex aberrant patients with adverse abnormalities *per se* are summarized to the CK+adv group (*n*=333) resulting from *n*=35 CK3+adv and *n*=298 CK4+adv patients. CK3+adv consists of complex aberrant karyotypes with three unrelated aberrations with at least one specific adverse-risk aberration, without HDK, without t(9;11) and CK4+adv consists of complex karyotypes with ⩾4 unrelated aberrations with at least one specific adverse-risk aberration, without HDK, without t(9;11).

The distinct cytogenetic features were considered with the following groups of complex aberrant patients: (a) HDK, (b) t(9;11), (c) complex karyotypes with three unrelated aberrations without specific adverse-risk aberrations, without HDK, without t(9;11) (CK3), (d) complex karyotypes with three unrelated aberrations with at least one specific adverse-risk aberration, without HDK, without t(9;11) (CK3+adv), (e) complex karyotypes with ⩾4 unrelated aberrations without specific adverse-risk aberrations, without HDK, without t(9;11) (CK4) and (f) complex karyotypes with ⩾4 unrelated aberrations with at least one specific adverse-risk aberration, without HDK, without t(9;11) (CK4+adv). Comprehensive flowcharts of the distinct groups of complex aberrant karyotypes are depicted in Figures 1a and b.

In order to investigate the influence of an MK in the complex aberrant situation, patients were divided into (a) patients with three unrelated aberrations without MK (CK3−MK), (b) patients with three unrelated aberrations with MK (CK3+MK), (c) patients with ⩾4 unrelated aberrations without MK (CK4−MK) and (d) patients with ⩾4 unrelated aberrations with MK (CK4+MK).

### Treatment protocols

Detailed treatment descriptions of the three trials were reported previously.^13, 17, 18^ In brief, the AML96 trial enrolled adult patients without age restriction, whereas the AML2003 trial included patients up to 60 years of age, and the AML60+ trial patients above the age of 60 years. Apart from double induction chemotherapy administered to patients aged ⩽60 years, all three protocols involved a risk-adapted consolidation strategy, including HLA-compatible related or unrelated allogeneic hematopoietic stem cell transplantation for intermediate-risk patients with a sibling donor and adverse-risk patients with a matched donor. In the AML2003 trial, patients were randomized up-front to undergo allogeneic hematopoietic stem cell transplantation early after induction chemotherapy-induced aplasia or during first remission in defined adverse-risk situations.^19^

### Statistical analysis

Complete remission was defined according to the standard consensus criteria.^20^ OS was measured from the date of entering the study to the date of event (death) or last follow-up and was reported for the whole cohort. The Kaplan–Meier method was used to estimate the probability for OS. Median OS were provided for all end points with 95% confidence intervals (CIs). The stratified log rank test was used for univariate comparison of OS. The stratification variable was the study generation.

To determine the prognostic influence of the distinct cytogenetic groups independent of age, WBC, serum lactate dehydrogenase levels at baseline, and type of AML (*de novo* AML, AML with preceding myelodysplastic syndrome, therapy-related AML) as covariates, a stratified multivariable Cox regression analysis for OS was performed. Stratification variable again was study generation. Because of its informative character, allogeneic hematopoietic stem cell transplantation was not censored. All statistical analyses were performed using SPSS version 19.0.1 (SPSS Inc, Chicago, IL, USA) and the R environment for statistical computing version 2.15.3.^21^

## Results

The following cytogenetic subgroups were analyzed for their influence on OS.

### Hyperdiploid karyotype

Out of 417 patients with ⩾3 aberrations, 20 patients displayed a pure HDK with a range of 49–80 chromosomes (median, 50 chromosomes) without other abnormalities. Nine patients with HDK had 3 trisomies and 11 patients had ⩾4 trisomies. The most frequent chromosomes involved in the formation of HDK were chromosomes 8, 4, 13, 9, 10, 21 and 22 (in decreasing frequency), present in at least more than 20% of all patients with HDK. Additionally, tetrasomies 4, 8, 13, 14, 20 and 21, each detected in 1–2 patients, as well as pentasomies 13, 21 and 22, each detected in one patient, were found.

**Figure 2:**
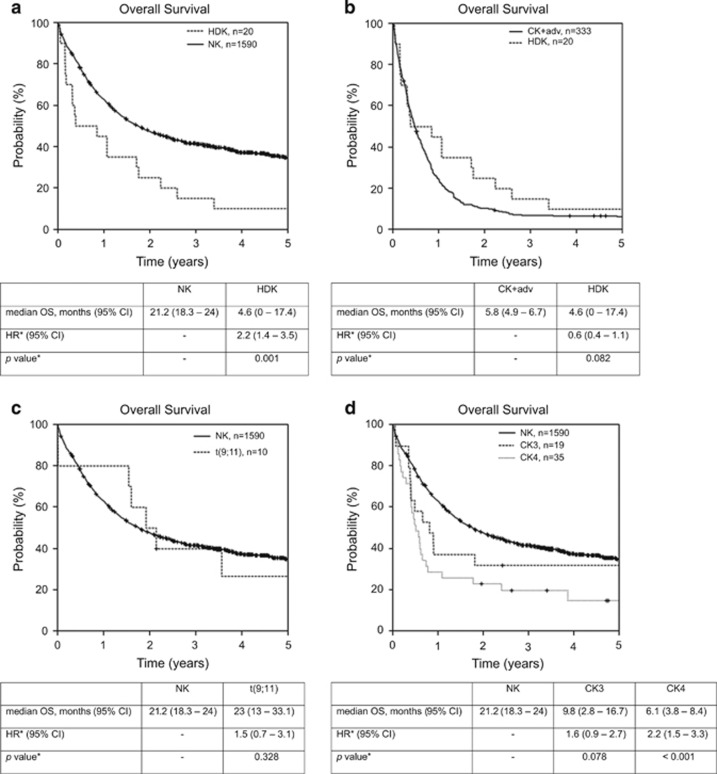
(**a**) Overall survival (OS) of patients with normal karyotype (NK) and with pure hyperdiploid karyotype (HDK) AML from the time of diagnosis. (**b**) OS of patients with HDK and with complex aberrant karyotype AML with ⩾3 aberrations (CK+adv) of which at least one aberration predicts an adverse risk *per se*, but without HDK and t(9;11) from the time of diagnosis. (**c**) OS of patients with NK and with complex aberrant karyotype with t(9;11)(p21–22;q23) from the time of diagnosis. (**d**) OS of patients with NK, with complex aberrant karyotype with three unrelated abnormalities but without HDK, t(9;11), and specific adverse-risk aberrations (CK3), with complex aberrant karyotype with ⩾4 unrelated abnormalities but without HDK, t(9;11), and specific adverse-risk aberrations (CK4). Median OS is depicted in the respective table. Cox regression (*, hazard ratio (HR), *P*-value) was performed applying age, WBC, LDH and the type of AML (AML with antecedent myelodysplastic syndrome and therapy-related AML) as co-variables. Abbreviation: LDH, lactate dehydrogenase.

The median OS for these patients was 4.6 months (95% CI, 0–17.4) (Figure 2a). The multivariable Cox regression including age, WBC, lactate dehydrogenase and type of AML showed that HDK was an independent prognostic factor for OS (HR 2.2; 95% CI, 1.4–3.5; *P*=0.001). There was no influence of the number of trisomies or tetrasomies on survival. Patients with three trisomies and patients with four or more trisomies/tetrasomies had a similar probabilities of OS (*P*=NS, data not shown). Furthermore, we compared HDK patients with patients with cytogenetic adverse-risk criteria according to the ELN/MRC classifications (Figure 2b). OS did not differ significantly (HR 0.6; 95% CI, 0.4–1.1; *P*=0.082). For the adverse control group (CK+adv, *n*=333) no further distinction between CK3+adv (*n*=35) and CK4+adv (*n*=298) was performed since HDK patients with 3 or ⩾4 aberrations had similar survival.

Additional data resulting from univariate comparisons (log rank test) regarding OS and data resulting from multivariable Cox regression analysis of these patients and the patients documented below are summarized in the supplement (Supplementary Tables S1 and S2).

### t(9;11) and other WHO recurrent cytogenetic abnormalities

The following WHO recurrent cytogenetic aberrations were detected within the complex aberrant karyotypes of the patients analyzed: t(9;11)(p21∼22;q23) (*n*=10 patients); t(6;9)(p23;q34) (*n*=3 patients); inv(3)(q21q26.2)/t(3;3)(q21;q26.2) (*n*=9 patients) and t(1;22)(p13;q13) (*n*=1 patient). Patients with t(9;11) had an OS similar to patients with NK with a median survival of 23 months (95% CI, 13–33.1) (Figure 2c) Interestingly, 8 out of the 10 patients had a karyotype grouped into the CK4 cohort and 2 patients had additional specific adverse-risk abnormalities.

### Complex karyotype with three independent aberrations (CK3) vs complex karyotype with four or more independent aberrations (CK4)

To delineate the best cutoff of complexity, we analyzed survival data from patients with 3 or ⩾4 independent aberrations (CK3, *n*=19 and CK4, *n*=35, respectively) who furthermore did not have any of the adverse-risk cytogenetic aberrations, without HDK, and without a t(9;11) (Figure 2d). CK4 patients without t(9;11) and without adverse-risk criteria had a significant inferior OS as compared with the control group. Multivariable Cox regression analysis confirmed these observations for OS showing that CK4 (without adverse-risk abnormalities, without t(9;11) and without HDK) is an independent adverse prognostic factor for OS in comparison with NK (HR, 2.2; 95% CI, 1.5–3.3; *P*<0.001). Interestingly, OS of CK3 patients without t(9;11) and the adverse-risk criteria was reduced only slightly (HR, 1.6; 95% CI, 0.9–2.7; *P*=0.078) while there was no significant effect for event-free survival at all (data not shown).

### MK/unique adverse-risk aberrations

**Figure 3:**
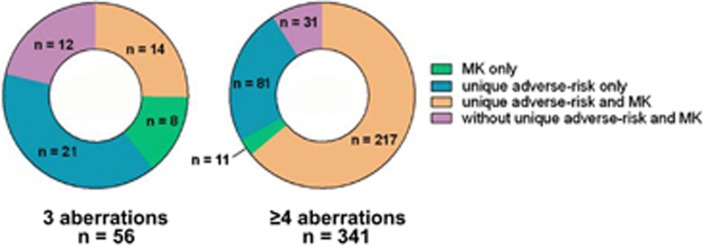
Distribution of karyotype abnormalities in patients with three abnormalities exclusive of an HDK and patients with ⩾4 abnormalities exclusive of an HDK. The karyotype abnormalities in these patients include monosomal karyotype (MK), unique adverse-risk risk karyotype only, unique adverse-risk risk karyotype in combination with MK, and patients without unique adverse-risk risk karyotype and MK. Patients with pure HDK are excluded.

**Figure 4:**
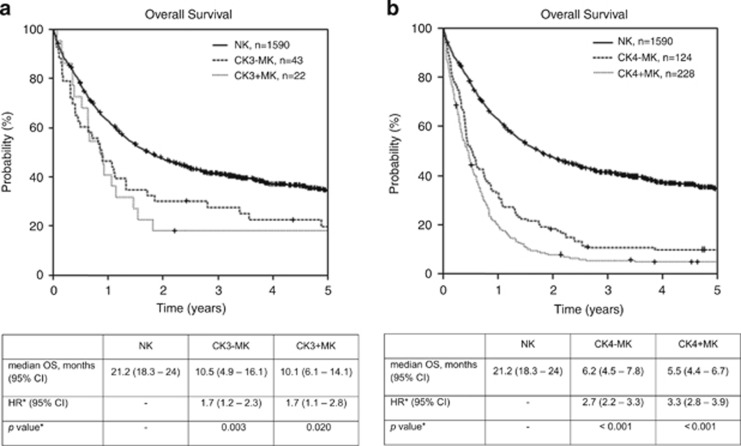
Overall survival (OS) from the time of diagnosis of patients with normal karyotype (NK), (**a**) with complex aberrant karyotype with three unrelated abnormalities but without MK (CK3−MK), with complex aberrant karyotype with three unrelated abnormalities and MK (CK3+MK), (**b**) with complex aberrant karyotype with ⩾4 unrelated abnormalities but without MK (CK4−MK), and with complex aberrant karyotype with ⩾4 unrelated abnormalities and MK (CK4+MK). Median OS are depicted in the respective tables. Cox regression (*, hazard ratio (HR), *P*-value) was performed applying age, WBC, LDH and the type of AML (AML with antecedent myelodysplastic syndrome and therapy-related AML) as co-variables. Abbreviation: LDH, lactate dehydrogenase.

Many patients shared the cytogenetic features MK and specific adverse-risk aberrations (Tables 2A and B and Figure 3). In the group of patients with three aberrations, 34% patients (*n*=22) had an MK, whereas in the group of patients with ⩾4 aberrations, 65% patients (*n*=228) had an MK, resulting in a total of 250 patients with MK. A total of 333 patients had specific adverse-risk aberrations (54%, *n*=35, in those patients with 3 aberrations and 82%, *n*=298 in those patients with ⩾4 aberrations). The frequency of chromosome 17p abnormalities in these groups was 11% and 38%, respectively. Of the patients belonging to the CK3+MK group (*n*=22), 64% had additional specific adverse-risk aberrations (*n*=14), whereas 95% (*n*=217) of the patients belonging to the CK4+MK group (*n*=228) had additional specific adverse-risk aberrations. Because MK is often caused by monosomy of chromosomes 5, 7 or 17, we investigated the frequencies of these monosomies in the MK group. A total of 45% of patients with CK3+MK as well as 66% patients with CK4+MK harbored at least one of the above monosomies. When investigating the influence of MK, all four subgroups that is, CK3*−*MK, CK3+MK, CK4−MK and CK4+MK, fared worse with regard to OS than the NK control group (Figures 4a and b).

The additional influence of specific adverse-risk aberrations on outcome was more pronounced in patients with CK4 as compared with patients with CK3 (CK3 vs CK3+adv and CK4 vs CK4+adv; Supplementary Figures S1a and b). Median OS in months were 9.8 (95% CI, 2.8–16.7) for CK3, 11 months (95% CI, 6–16.1) for CK3+adv, 6.1 months (95% CI, 3.8–8.4) for CK4 and 5.4 months (95% CI, 4.5–6.2) for CK4+adv, respectively. The hazard ratios for the risk of death in these groups compared with NK patients in the multivariable analysis were 1.6 for CK3 (95% CI, 0.9–2.7; *P*=0.085), 1.6 for CK3+adv (95% CI, 1.1–2.3; *P*=0.010), 2.3 for CK4 (95% CI, 1.6–3.3; *P*<0.001), and 3.3 for CK4+adv (95% CI, 2.8–3.8; *P*<0.001).

## Discussion

Our analysis of the prognostic role of karyotype complexity (3 vs ⩾4) in adult AML patients demonstrates that a higher number of patients have ⩾4 (84%) aberrations than 3 (16%) aberrations. Patients with ⩾4 aberrations clearly fare worse than NK patients, which could be demonstrated for the distinct groups CK4, CK4+adv, CK4−MK and CK4+MK. Of additional unfavorable influence on OS was the existence of unique adverse-risk aberrations (risk of death in comparison with NK patients for CK4 HR, 2.3; 95% CI, 1.6–3.3 vs CK4+adv HR, 3.3; 95% CI 2.8–3.8) or an MK (CK4−MK HR, 2.7; 95% CI, 2.2–3.3 vs CK4+MK HR, 3.3; 95% CI, 2.8–3.9). Patients with three unrelated aberrations had a worse outcome than NK patients, too. However, the effect was less impressive than in patients with ⩾4 aberrations.

The MRC data demonstrated that the level of karyotype complexity has little impact on the outcome in patients already having at least one of the independent abnormalities conferring favorable or adverse risk. Additionally, the MRC reported that in patients lacking any of these independent adverse-risk abnormalities, the presence of ⩾4 unrelated changes was found to provide the most informative cutoff, predicting a significantly inferior prognosis.^5^

The ELN classification scheme allocated patients with the recurring aberration t(9;11)(p22;q23) to the intermediate-II genetic risk group.^1^ Our data confirm this stratification showing that the t(9;11)(p21–22;q23) confers an intermediate risk even with an accompanying complex karyotype.

Our study implies that AML patients with an HDK, specifically those without additional monosomies or structural aberrations, should be allocated to an adverse-risk category because of the significant influence on survival in comparison with NK patients (OS HR, 2.2; 95% CI, 1.4–3.5; *P*=0.001). Recently, the impact of hyperdiploidy in AML patients was published independently, emphasizing the impact of this category.^6^ Although the authors identified a similar, obviously non-random pattern of chromosomal gains comparable to our data, they applied a different approach by also including patients with monosomies in their HDK group with numerical changes, whereas we addressed the impact of pure hyperdiploidy separately without including patients with structural abnormalities and patients with loss of chromosomes. An analysis of the French *Groupe Francophone de Cytogenetique Hematologique* investigated 38 AML patients with high HDKs restricted to karyotypes with only high hyperdiploidy with 49 or more chromosomes.^7^ Because of the inclusion of children in the analysis by Luquet *et al.*, a different methodology and different statistical methods being applied, a direct comparison with our results is not possible.

To date, it is not clear whether additional monosomies or structural aberrations occur earlier in the development of AML or whether one or the other abnormality acts as the driver or the passenger aberration. Although gains of additional chromosomes appear to represent the result of clonal evolution caused by the failure of the mitotic machinery rather than an initiating event in AML, we suggest classifying patients with pure HDK as a distinct category, excluding monosomies and structural abnormalities.

The introduction of the MK category by Breems *et al.* offered the application of a further criterion in risk stratification of AML patients showing that MK identifies a subset of patients with very poor prognosis, which has been confirmed by other groups.^5, 8, 22^ Our study, which was restricted to the distinct population of complex aberrant patients, showed a relevant influence on prognosis for the presence of an MK only in the CK4 situation with an increased risk of death for patients with CK4+MK as compared with patients with CK4−MK. In patients with CK3−MK or with CK3+MK the risk of death was superimposable. Additionally, our data confirm that distinct cytogenetic features that accompany other abnormalities have a strong influence on outcome and must be considered independently, for example, patients with t(9;11) conferring intermediate risk.

A consistent definition of adverse-risk complex aberrant karyotype AML appears to be warranted. Here, we confirm a karyotype with ⩾4 aberrations and a pure HDK as impressive adverse-risk abnormalities in AML. Patients with three unrelated aberrations fare worse than NK, too, but with an OS classifying between the ⩾4 patients and the intermediate NK. This is an important finding that may help to stratify patients to individual optimized treatment strategies and may therefore lead to improved individual survival prognostication. Therefore, based on our findings, we suggest the following re-classification of cytogenetic risk: (1) favorable risk: CBF-AML; (2) intermediate risk: normal karyotype, t(9;11); (3) adverse risk: three aberrations without specific adverse-risk abnormalities, without HDK; (4) very adverse risk: ⩾4 aberrations, HDK, specific adverse-risk abnormalities, as defined by the ELN and MRC.

## Figures and Tables

**Table 1A:** Patient characteristics

	*NK*	*HDK*	*t(9;11)*	*CK3*	*CK4*	*CK+adv*
	n=*1590*	n=*20*	n=*10*	n=*19*	n=*35*	n=*333*
Median age (range)	56 (17–87)	60 (37–76)	40 (29–60)	56 (33–78)	56 (18–80)	61 (15–82)
Gender, male (%)	741 (47)	10 (50)	4 (40)	11 (58)	17 (49)	172 (52)
*De novo* AML (%)	1318 (83)	16 (80)	9 (90)	12 (63)	26 (74)	221 (66)
Median WBC (Gpt/l), range	16 (0.2–453)	12 (0.9–192)	24 (1–212)	10 (0.8–73)	7.5 (0.6–468)	4.1 (0.2–197)
Median LDH (U/l), range	427 (10–16 560)	513 (100–3058)	881 (276–2997)	261 (27–5489)	660 (93–7938)	362 (97–4160)

**Table 1B:** Patient characteristics

	*CK3+MK*	*CK3−MK*	*CK4+MK*	*CK4−MK*
	n=*22*	n=*43*	n=*228*	n=*124*
Median age (range)	63 (32–75)	62 (24–82)	61 (15–82)	58 (18–81)
Gender, male (%)	12 (54)	24 (56)	118 (52)	60 (48)
*De novo* AML (%)	14 (64)	30 (70)	149 (65)	91 (73)
Median WBC (Gpt/l), range	7 (0.8–98)	11 (0.6–156)	4 (0.2–468)	5 (0.4–212)
Median LDH (U/l), range	406 (135–2540)	371 (27–5489)	361 (97–7938)	414 (93–3946)

Abbreviations: NK, normal karyotype (control group); HDK, hyperdiploid karyotype; CK3, complex aberrant patients with three unrelated abnormalities without HDK, without t(9;11), and without adverse-risk abnormalities; CK4, complex aberrant patients with four or more unrelated abnormalities without HDK, without t(9;11), and without adverse-risk abnormalities; CK+adv, complex aberrant patients with three or more aberrations of which at least one aberration was at specific adverse risk *per se* with exclusion of patients with t(9;11) or HDK; CK3+MK, patients with MK and with three unrelated aberrations; CK3*−*MK, patients with three unrelated aberrations without MK; CK4+MK, patients with four or more unrelated aberrations with MK; CK4−MK, patients with four or more unrelated aberrations without MK; WBC, white blood count; LDH, lactate dehydrogenase.

**Table 2A:** Karyotype details in patients with 3 or ⩾4 aberrations without specific consideration of other abnormalities (see also Supplementary Figures 1A and B)

	*3 aberrations*n=*65*	*⩾4 aberrations*n=*352*
Hyperdiploid karyotype (%)	9 (14)	11 (3)
Adverse-risk abnormality (%)	35 (54)	298 (82)
abnl(17p) (%)	7 (11)	135 (38)
Monosomal karyotype (%)	22 (34)	228 (65)

Abbreviation: abnl(17p), abnormality of chromosome 17p.

**Table 2B:** Karyotype details in patients with 3 or ⩾4 aberrations and with a monosomal karyotype

	*CK3+MK*	*CK4+MK*
	n=*22*	n=*228*
Monosomal karyotype without adverse-risk abnormality (%)	8 (36)	11 (5)
Monosomal karyotype with −5, −7 or −17 (%)	10 (45)	151 (66)

Abbreviations: CK3+MK, patients with a monosomal karyotype and a complex karyotype with three unrelated aberrations; CK4+MK, patients with a monosomal karyotype and a complex karyotype with ⩾4 unrelated aberrations.
