# Supplementary material for: Karyotype complexity and prognosis in acute myeloid leukemia
Source: Blood Cancer J. 2016 Jan 15;6(1):e386–. doi: 10.1038/bcj.2015.114 (PMC4742631; doi:10.1038/bcj.2015.114)
Supplement: Supplementary Table 1 [file bcj2015114x1.docx]

**Supplemental Tables 1A and 1B.**

Supplemental Table 1A. *p*-values resulting from univariate comparisons (log-rank test) regarding OS of patients with NK, CK3-MK, CK3+MK, CK4-MK, and CK4+MK (see also Figures 4A and 4B).

|  | **NK** | **MK3-MK** | **MK3+MK** | **MK4-MK** | **MK4+MK** |
| --- | --- | --- | --- | --- | --- |
| **NK** | - | .01 | .066 | < .001 | < .001 |
| **MK3-MK** | - | - | .901 | .030 | - |
| **MK3+MK** | - | - | - | - | .002 |
| **MK4-MK** | - | - | - | - | .018 |

Supplemental Table 1B. *p*-values resulting from univariate comparisons (log-rank test) regarding OS of patients with NK, CK3, CK3+adv, CK4, and CK4+adv (see also Figure 2D, supplemental Figures 1A and 1B).

|  | **NK** | **CK3** | **CK3+adv** | **CK4** | **CK4+adv** |
| --- | --- | --- | --- | --- | --- |
| **NK** | - | .257 | .005 | < .001 | < .001 |
| **CK3** | - | - | .479 | .121 | - |
| **CK4** | - | - | - | - | .199 |
