# Supplementary material for: Karyotype complexity and prognosis in acute myeloid leukemia
Source: Blood Cancer J. 2016 Jan 15;6(1):e386–. doi: 10.1038/bcj.2015.114 (PMC4742631; doi:10.1038/bcj.2015.114)
Supplement: Supplementary Table 2 [file bcj2015114x2.docx]

**Supplemental Tables 2**. Multivariable Cox regression analysis for OS with age, WBC, serum LDH, and type of AML (sAML or tAML) as covariates.

**Table 2A.** Comparison of distinct cytogenetic groups with NK patients (control group).

| **Parameter** | **Hazard-Ratio** | **95% CI** | ***p*-value** | **Adjusting variables: HR, (95% CI), *p*-value** |
| --- | --- | --- | --- | --- |
| normal karyotype  HDK | reference  2.2 | (1.4 – 3.5) | 0.001 | Age: 1.03, (1.03 – 1.04), < 0.001; WBC: 1.2 (1.0 – 1.3), 0.02; LDH: 1.5, (1.2 – 1.9), 0.002; tAML: 1.4, (0.9 – 2.1), 0.101; sAML: 1.4, (1.2 – 1.7), <0.001 |
| normal karyotype  t(9;11)  CK3  CK4 | reference  1.5  1.6  2.2 | (0.7 – 3.1)  (0.9 – 2.7)  (1.5 – 3.3) | 0.328  0.078  < 0.001 | Age: 1.03, (1.03 – 1.04), < 0.001; WBC: 1.1, (1.0 – 1.3), 0.022; LDH: 1.6, (1.2 – 2.0), <0.001; tAML: 1.4, (1.0 – 2.1), 0.082; sAML: 1.3, (1.1 – 1.6), 0.001 |
| normal karyotype  CK3  CK3+adv | reference  1.6  1.6 | (0.9 – 2.7)  (1.1 – 2.3) | 0.085  0.010 | Age: 1.03, (1.03 – 1.04), < 0.001; WBC: 1.2, (1.0 – 1.3), 0.012; LDH: 1.5, (1.2 – 1.9), 0.001; tAML: 1.4, (0.9 – 2.1), 0.089; sAML: 1.4, (1.2 – 1.6), < 0.001 |
| normal karyotype  CK4  CK4+adv | reference  2.3  3.3 | (1.6-3.3)  (2.8 – 3.8) | < 0.001  < 0.001 | Age: 1.03, (1.03 – 1.04), < 0.001; WBC: 1.2, (1.0 – 1.3), 0.012; LDH: 1.5, (1.2 – 1.9), 0.001; tAML: 1.4, (0.9 – 2.1), 0.022; sAML: 1.4, (1.2 – 1.6), < 0.001 |
| normal karyotype  CK3-MK  CK3+MK  CK4-MK  CK4+MK | reference  1.7  1.7  2.7  3.3 | (1.2 – 2.3)  (1.1 – 2.8)  (2.2 – 3.3)  (2.8 – 3.9) | 0.003  0.020  < 0 .001  < 0 .001 | Age: 1.03, (1.03 – 1.04), < 0.001; WBC: 1.2, (1.1 – 1.3), 0.002; LDH: 1.6, (1.3 – 2.0), < 0.001; tAML: 1.4, (1.1 – 1.9), 0.014; sAML: 1.3, (1.1 – 1.5), < 0.001 |

**Table 2B.** Comparison of two distinct cytogenetic groups, each with distinct complex aberrant features.

| **Parameter** | **Hazard-Ratio** | **95% CI** | ***p*-value** | **Adjusting variables: HR, (95% CI), *p*-value** |
| --- | --- | --- | --- | --- |
| CK+adv  HDK | reference  0.6 | (0.4 – 1.1) | 0.082 | Age: 1.04, (1.03 – 1.05), < 0.001; WBC: 1.4 (1.1 – 1.7), 0.008; LDH: 2.1, (1.4 – 3.2), < 0.001; tAML: 1.6, (1.1 – 2.4), 0.032; sAML: 1.2, (0.9 – 1.6), 0.114 |

Abbreviations: tAML, therapy-related AML; sAML, AML with preceding MDS; NK, normal karyotype (control group); HDK, hyperdiploid karyotype; CK3, complex aberrant patients with three unrelated abnormalities without HDK, without t(9;11), and without adverse risk abnormalities; CK4, complex aberrant patients with four or more unrelated abnormalities without HDK, without t(9;11), and without adverse risk abnormalities; CK+adv, complex aberrant patients with three or more aberrations of which at least one aberration was at specific adverse risk per se with exclusion of patients with t(9;11) or HDK; CK3+adv, complex aberrant patients with three unrelated aberrations of which at least one aberration was at specific adverse risk per se with exclusion of patients with t(9;11) or HDK; CK4+adv complex aberrant patients with four or more aberrations of which at least one aberration was at specific adverse risk per se with exclusion of patients with t(9;11) or HDK; CK3+MK, patients with monosomal karyotype (MK) and with 3 unrelated aberrations; CK3-MK, patients with 3 unrelated aberrations without MK; CK4+MK, patients with four or more unrelated aberrations with MK; CK4-MK, patients with four or more unrelated aberrations without MK; WBC, white blood count; LDH, lactate dehydrogenase.
