# Supplementary material for: Karyotype complexity and prognosis in acute myeloid leukemia
Source: Blood Cancer J. 2016 Jan 15;6(1):e386–. doi: 10.1038/bcj.2015.114 (PMC4742631; doi:10.1038/bcj.2015.114)
Supplement: Supplementary Figures [file bcj2015114x3.docx]

**Supplemental Figure 1A.** Overall Survival of patients with NK, CK3, and CK3+adv from time of diagnosis

0

1

2

3

4

5

0

20

40

60

80

100

**Overall Survival**

Time (years)

Probability (%)

CK3+adv, n=35

NK, n=1590

CK3, n=19

|  | NK | CK3 | CK3+adv |
| --- | --- | --- | --- |
| median OS, months (95% CI) | 21.2 (18.3 – 24) | 9.8 (2.8 – 16.7) | 11 (6 – 16.1) |
| HR* (95% CI) | - | 1.7 (1 – 2.9) | 1.7 (1.2 – 2.4) |
| *p* value* | - | .047 | .005 |

**Supplemental Figure 1B.** Overall Survival of patients with NK, CK4, and CK4+adv from time of diagnosis

0

1

2

3

4

5

0

20

40

60

80

100

**Overall Survival**

Time (years)

Probability (%)

CK4+adv, n=298

NK, n=1590

CK4, n=35

|  | NK | CK4 | CK4+adv |
| --- | --- | --- | --- |
| median OS, months, (95% CI) | 21.2 (18.3 – 24) | 6.1 (3.8 – 8.4) | 5.4 (4.5 – 6.2) |
| HR* (95% CI) | - | 2.4 (1.7 – 3.5) | 3.3 (2.9 – 3.9) |
| *p* value* | - | < .001 | < .001 |
