# Supplementary material for: Karyotype complexity and prognosis in acute myeloid leukemia
Source: Blood Cancer J. 2016 Jan 15;6(1):e386–. doi: 10.1038/bcj.2015.114 (PMC4742631; doi:10.1038/bcj.2015.114)
Supplement: Supplementary Figure Legends [file bcj2015114x4.docx]

**Figure Legends – Supplemental Figures**

**Supplemental Figures 1A and 1B**

Overall Survival (OS) from time of diagnosis of patients with normal karyotype (NK); with complex aberrant karyotype with 3 unrelated abnormalities but without HDK, t(9;11), and specific adverse risk aberrations (CK3), with complex aberrant karyotype with 3 unrelated abnormalities of which at least one aberration predicts an adverse risk *per se* (CK3+adv) but without HDK and t(9;11), complex aberrant karyotype with ≥4 unrelated abnormalities but without HDK, t(9;11), and without specific adverse risk aberrations (CK4), and with complex aberrant karyotype with ≥4 unrelated abnormalities of which at least one aberration predicts an adverse risk *per se* (CK4+adv) but without HDK and t(9;11). Median OS is depicted in the respective tables below. Cox regression (*, hazard ratio HR, *p*-value) was performed applying age, WBC, LDH, and the type of AML (AML with antecedent MDS and therapy-related AML) as co-variables.
